# Urinary ATP may be a biomarker of interstitial cystitis/bladder pain syndrome and its severity

**DOI:** 10.17305/bb.2023.9694

**Published:** 2024-02-01

**Authors:** Yanyuan Wu, Yedie He, Jun Qi, Song Wang, Zongping Wang

**Affiliations:** 1 Department of Urology, Cancer Hospital of the University of Chinese Academy of Sciences (Zhejiang Cancer Hospital), Hangzhou, China; 2 Institute of Cancer and Basic Medicine (IBMC), Chinese Academy of Sciences, Hangzhou, China; 3 Department of Urology, Xinhua Hospital Affiliated to Shanghai Jiao Tong University, School of Medicine, Shanghai, China

**Keywords:** Lower urinary tract symptoms (LUTS), lower abdominal pain, interstitial cystitis/bladder pain syndrome (IC/BPS), adenosine triphosphate (ATP)

## Abstract

Urinary tract cells respond to bladder distension by releasing adenosine triphosphate (ATP). Patients with interstitial cystitis/bladder pain syndrome (IC/BPS) exhibit elevated urinary ATP levels compared to asymptomatic controls. This study aimed to evaluate the potential of urinary ATP as a non-invasive biomarker for IC/BPS and its correlation with symptom severity. We included 56 patients diagnosed with IC/BPS and 50 asymptomatic controls. Urine samples were collected from both groups. Urinary ATP levels were quantified using the luciferin–luciferase bioluminescence method. The severity of IC/BPS symptoms was assessed using the visual analog score (VAS), Interstitial Cystitis Symptom Index (ICSI), and Interstitial Cystitis Problem Index (ICPI) from the O’Leary–Sant score. We specifically examined the correlation between symptom scores and urinary ATP levels in IC/BPS patients. Urinary ATP levels were significantly higher in IC/BPS patients compared to the control group (*P* < 0.0001). There was a significant positive correlation between urinary ATP concentrations and VAS, ICPI, and ICSI scores among IC/BPS patients (*P* < 0.0001). The threshold value for ATP concentration was set at 56.6 nM, with an area under the receiver operating characteristic (ROC) curve of 0.811 (95% CI 0.730–0.892). Our findings indicate that IC/BPS patients excrete elevated amounts of ATP in their urine. This suggests that urinary ATP might serve as a non-invasive biomarker for IC/BPS, with a predictive potential in terms of symptom severity.

## Introduction

Interstitial cystitis/bladder pain syndrome (IC/BPS) is characterized by persistent pelvic pain and lower urinary tract symptoms (LUTS). While current epidemiological data on IC/BPS is limited, it is widely acknowledged that the condition is more commonly observed in women [1]. Additionally, IC/BPS has been found to be closely linked to factors, such as age, race, and other variables [2, 3]. The European Association of Urology (EAU) outlines significant challenges in diagnosing IC/BPS. According to the EAU, the diagnosis of IC/BPS primarily relies on symptom assessment and the use of cystoscopy [4]. The O’Leary–Sant questionnaire, with its favorable specificity and sensitivity, stands out as a valuable diagnostic instrument for IC/BPS identification [5]. The visual analog score (VAS) demonstrates enhanced sensitivity in detecting pain among patients. Nevertheless, it is important to note that the objective evaluation of patients’ pain levels using scales remains limited due to the lack of standardized criteria. Cystoscopy, a procedure that is both time consuming and invasive, imposes significant pain and financial burden on certain patients. Given these challenges, there is a pressing need for the development of a non-invasive and cost-effective test or biomarker that can accurately identify the presence and gauge the severity of IC/BPS.

The conventional understanding of the bladder urothelium was that it served solely as a passive barrier. However, recent studies have indicated that it has the ability not just to detect but also to secrete signaling molecules, such as adenosine triphosphate (ATP), acetylcholine (Ach), and prostaglandin E2 (PGE2) in response to various physical or chemical stimuli [6, 7]. ATP acts as a stimulant, activating the P2X purinoreceptor 3 (P2X3) located on afferent fibers, consequently initiating the voiding reflex [8, 9]. Under specific pathological circumstances, the urothelium exhibits variations in the release of ATP into the urinary lumen. For instance, patients with benign prostatic hyperplasia (BPH) demonstrate alterations in urinary ATP concentrations [10], as do those with bladder infections or inflammation [11]. Hence, urinary ATP is regarded as a biomarker for the aforementioned conditions.

Previous research has indicated that individuals with IC/BPS exhibit elevated urinary ATP levels compared to asymptomatic controls. However, it is worth noting that these studies did not utilize a questionnaire to assess the severity of IC/BPS symptoms in patients [12]. In this research, we investigated the potential of urinary ATP as a non-invasive biomarker for IC/BPS. Additionally, we explored the relationship between the disease’s severity and urinary ATP levels by analyzing the correlation between the VAS, Interstitial Cystitis Symptom Index (ICSI), Interstitial Cystitis Problem Index (ICPI), and urinary ATP.

## Materials and methods

### Patients

**Figure 1.**
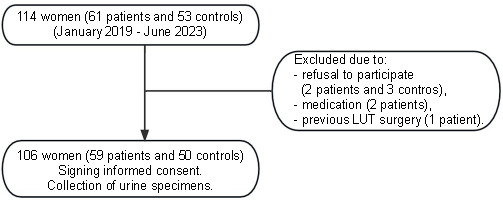
**Flow diagram of patients’ selection.** LUT: Lower urinary tract.

Female IC/BPS patients from the Department of Urology at our hospital were randomly recruited between January 2019 and June 2023. IC/BPS was diagnosed based on the criteria from the National Institute of Diabetes and Digestive and Kidney Disease (NIDDK), which requires patients to exhibit symptoms of pelvic/lower abdominal pain associated with bladder filling, LUTS (urination > 11 times/24 h, nocturia > 2 times/night), and either Hunner’s ulcer or a glomerulation spotted during cystoscopy. Inclusion criteria were as follows: (1) subjects voluntarily signed the informed consent form; (2) subjects were aged between 18 and 70 years, conscious, compliant, capable of clearly expressing their personal feelings, and able to complete the symptom questionnaire independently; (3) patients in the IC/BPS group strictly met the diagnostic criteria; and (4) routine blood, urine, liver, and kidney function indicators were within normal ranges. Exclusion criteria were as follows: (1) patients who had undergone treatment and experienced symptoms relief within the last six months; (2) patients with associated conditions, such as bladder stones, ureteral stones, chemical cystitis, radiation cystitis, urological tumors, urinary tract infections (UTIs), reproductive system inflammatory diseases, endometriosis, etc.; (3) patients who had consumed opioids or stronger drugs. Nonsteroidal anti-inflammatory drugs (NSAIDs) should have been discontinued for at least six months; and (4) special populations such as pregnant women. Initially, 61 patients were considered, but five were excluded (two patients due to refusal to participate, two patients because they were on analgesic medication, and one patient with a history of lower urinary tract surgery), leaving 56 patients with an average age of 51.45 ± 11.62 years. From the Health Screening Center of Xinhua Hospital, Shanghai Jiao Tong University School of Medicine, 53 asymptomatic female volunteers were initially selected. After excluding three who declined to participate, 50 volunteers (aged 53.22 ± 12.65 years) remained (Figure 1). Exclusion criteria for the volunteers encompassed any UTI, bladder outlet obstruction (BOO), overactive bladder (OAB), malignancy, neurological diseases, and other LUTS.

The collected basic clinical data included age, height, weight, menstrual status, the presence of hypertension, diabetes mellitus, coronary artery disease, routine blood test, routine urine test, and blood biochemistry tests. Additionally, VAS pain scores, ICSI scores, and ICPI scores, obtained before initiating any treatment, were also documented.

### Measurement of urinary ATP and creatinine

Urine samples were collected prior to cystoscopy. To mitigate the influence of the urine collection time on samples, we established a specific urine collection window from 8:00 a.m. to 9:00 a.m. Patients were instructed to consume 250 mL of water after completely emptying their bladders. Mid-stage urine samples were then collected at designated intervals of 10, 20, and 30 min from both IC/BPS patients and volunteers. Upon collection, the urine samples were immediately placed on ice and treated with adenylyl-imidodiphosphate. To prevent ATP degradation, these samples were analyzed within 30 min of collection. An assayer, who was blinded to the sample source, conducted the measurements of ATP and creatinine. Initially, the samples were centrifuged at 500 rpm for 5 min to remove dead cells, and the resulting supernatant was used to determine the ATP and creatinine concentrations.

Following the manufacturer’s guidelines (Sigma-Aldrich, St. Louis, MO, USA), 100 µL of the ATP assay working solution was introduced into each assay well. After a 5-min incubation period to eliminate any background ATP, 20 µL of the sample was individually added to each well and mixed thoroughly. The ATP concentration was then determined by measuring the luminescence value utilizing an enzyme marker equipped with a luminometer function, in conjunction with a standard curve. Urinary creatinine levels were quantified using a commercially available kit from Roche Diagnostics GmbH (Mannheim, Germany).

### Ethical statement

This study, along with all associated procedures, received approval from the Ethics Committee of Xinhua Hospital, Shanghai Jiao Tong University School of Medicine (Ethics No. XHEC-C-2021-106-1). All patients signed an informed consent form prior to any examination. The study adhered to the principles outlined in the Declaration of Helsinki.

### Statistical analysis

Experimental results were statistically analyzed using the SPSS 26.0 and GraphPad Prism 9.0 data analysis software. When the measurement data followed a normal distribution, comparisons between the two groups utilized the two independent-sample *t*-tests. For non-normally distributed data, the non-parametric Mann–Whitney *U* test was applied. Count data were analyzed using the chi-square test. To determine its diagnostic value, urinary ATP concentration underwent analysis by the receiver operating characteristic (ROC), and the associated area under the curve (AUC) was computed. The correlation between urinary ATP concentration and clinical symptom scores was examined using the Pearson analysis. Correlation strength was categorized as follows: |*r*| < 0.5 represented low correlation, 0.5 ≤ |*r*| < 0.8 represented moderate correlation, and |*r*| ≥ 0.8 represented high correlation. A *P* value < 0.05 was deemed statistically significant. The significance levels are denoted as: *P* < 0.05 (*); *P* < 0.01 (**); *P* < 0.001 (***); *P* < 0.0001 (****).

## Results

### Urinary ATP levels are elevated in IC/BPS patients compared to asymptomatic controls

**Figure 2.**
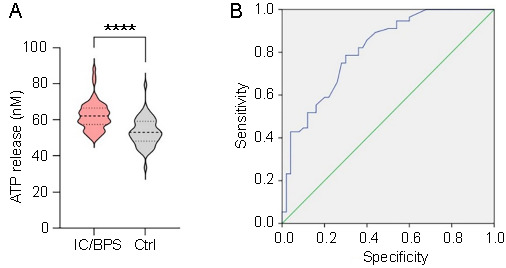
**Diagnostic value of urinary ATP in IC/BPS patients.** (A) Comparison of urinary ATP concentrations between IC/BPS patients and asymptomatic controls. There is a significant difference between the two groups (*P* < 0.0001). (B) ROC curve for urinary ATP concentration. The AUC value for ATP was 0.828, indicating significance (*P* < 0.0001). ATP: Adenosine triphosphate; IC/BPS: Interstitial cystitis/bladder pain syndrome; Ctrl: Control; ROC: Receiver operating characteristic; AUC: Area under the curve.

The clinical characteristics of IC/BPS patients (*n* ═ 56) and asymptomatic controls (*n* ═ 50) are detailed in Table 1. Both age and body mass index (BMI) showed no significant differences between the two groups. Specifically, the ages were 51.45 ± 11.62 years for the IC/BPS group vs 53.22 ± 12.65 years for the control group (*P* ═ 0.454), and the BMI values were 24.35 ± 3.96 kg/m^2^ vs 23.32 ± 4.29 kg/m^2^, respectively (*P* ═ 0.201). However, IC/BPS patients had significantly higher scores on the VAS (5.04 ± 1.93 vs 0.50 ± 0.79; *P* < 0.0001), ICSI (16.21 ± 2.64 vs 0.50 ± 0.79; *P* < 0.0001), and ICPI (15.80 ± 2.49 vs 0.58 ± 0.76; *P* < 0.0001) compared to the asymptomatic volunteers. Urinary ATP concentrations were also significantly higher in IC/BPS patients in comparison to the normal controls, with values being 62.99 ± 7.97 nM vs 53.26 ± 7.82 nM, respectively (*P* < 0.0001) (Figure 2A). There were no significant differences in urinary creatinine levels between the two groups (94.92 ± 8.06 mg/dL for the IC/BPS group vs 92.91 ± 10.03 mg/dL for controls; *P* ═ 0.273).

**Table 1:** Clinical characteristics of IC/BPS patients and asymptomatic controls

**Parameters**	**Patients** **(*n* ═ 106)**	**IC/BPS group** **(*n* ═ 56)**	**Control group** **(*n* ═ 50)**	***P* value**
Age (years)	52.28 ± 12.09	51.45 ± 11.62	53.22 ± 12.65	0.454
BMI (kg/m^2^)	23.86 ± 4.14	24.35 ± 3.96	23.32 ± 4.29	0.201
Serum leukocytes (× 10^9^/L)	5.89 ± 1.58	6.06 ± 1.65	5.72 ± 1.49	0.265
Serum neutrophils (× 10^9^/L)	3.56 ± 1.35	3.63 ± 1.50	3.49 ± 1.17	0.605
Serum platelets (× 10^9^/L)	191.90 ± 46.59	196.34 ± 46.20	186.92 ± 46.98	0.301
Hypertension				0.542
Yes	12	5	7	
No	94	51	43	
Diabetes				0.418
Yes	6	4	2	
No	100	52	48	
Coronary artery disease				0.443
Yes	7	5	2	
No	99	51	48	
Menopause				0.693
Yes	42	19	21	
No	64	37	29	
Urinary ATP (nM)	58.122 ± 9.25	62.99 ± 7.97	53.26 ± 7.82	<0.0001
Urinary creatinine (mg/dL)	93.91 ± 9.11	94.92 ± 8.06	92.91 ± 10.03	0.273
VAS	2.89 ± 2.72	5.04 ± 1.93	0.50 ± 0.79	<0.0001
ICSI	8.95 ± 7.99	16.21 ± 2.64	0.82 ± 1.04	<0.0001
ICPI	8.62 ± 7.86	15.80 ± 2.49	0.58 ± 0.76	<0.0001

The numbers represent mean ± standard deviation or *n*. IC/BPS: Interstitial cystitis/bladder pain syndrome; BMI: Body mass index; ATP: Adenosine triphosphate; VAS: Visual analog score; ICSI: Interstitial cystitis symptom index; ICPI: Interstitial cystitis problem index.

**Figure 3.**
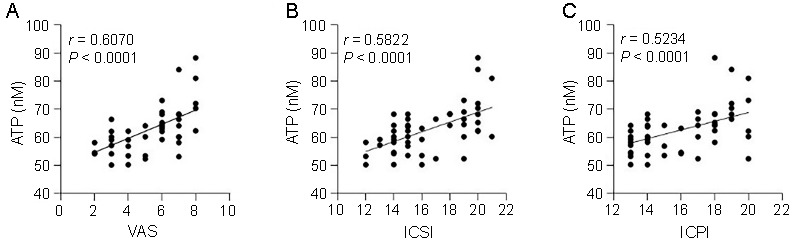
**Linear regression analysis of urinary ATP concentration and IC/BPS symptom scores in IC/BPS patients**. Urinary ATP of IC/BPS patients showed a significant positive correlation with (A) VAS (*r* ═ 0.6070; *P* < 0.0001), (B) ICSI (*r* ═ 0.5822; *P* < 0.0001), and (C) ICPI (*r* ═ 0.5234; *P* < 0.0001). ATP: Adenosine triphosphate; IC/BPS: Interstitial cystitis/bladder pain syndrome; VAS: Visual analog score; ICSI: Interstitial cystitis symptom index; ICPI: Interstitial cystitis problem index.

To evaluate the diagnostic efficacy of urinary ATP for IC/BPS, an ROC curve was constructed to analyze the urinary ATP concentration across all participants (Figure 2B). The AUC value for ATP stood at 0.811 (95% CI 0.730–0.892; *P* < 0.0001). The optimal threshold for ATP concentration, as determined through the ROC analysis, utilizing the maximal Youden index, was found to be 56.6 nM.

### Correlation of urinary ATP with symptom scores in IC/BPS patients

Based on the aforementioned findings, this study utilized the urinary ATP concentrations to investigate potential correlations between IC/BPS patients and symptom scores. Pearson correlation analysis was conducted, with the results summarized in Figure 3. As anticipated, VAS (*r* ═ 0.6070; *P* < 0.0001; Figure 3A), ICSI (*r* ═ 0.5822; *P* < 0.0001; Figure 3B), and ICPI (*r* ═ 0.5234; *P* < 0.0001; Figure 3C) all exhibited positive correlations with urinary ATP levels in IC/BPS patients.

## Discussion

Cystoscopy is performed under local anesthesia at our institution. For the safety of patients, biopsy is not a standard procedure, making the obtaining of bladder tissue from IC/BPS patients challenging. This study builds on previous observations that urinary ATP levels are elevated in IC/BPS patients compared to asymptomatic controls [12]. In this study, we assessed symptom severity in IC/BPS patients using various scales. We found a positive correlation between urinary ATP concentration and scores on the VAS, ICSI, and ICPI. To differentiate between IC/BPS and non-IC/BPS patients, we concluded a threshold of ATP at > 56.6 nM. The area under the ROC curve was calculated to be 0.811 (95% CI 0.730–0.892; *P* < 0.0001). These results indicate that urinary ATP levels could serve as a non-invasive biomarker for IC/BPS and are associated with symptom severity.

The participants in our research were exclusively females due to the higher prevalence of IC/BPS in women. It has been reported that approximately 12% of women experience this condition at some point in their lives. However, the prevalence of IC/BPS in men has often been underestimated [13]. The menstrual status across participants in both groups was found to be similar. Furthermore, our study did not find a significant association between age and urinary ATP concentration, neither in IC/BPS patients (*P* ═ 0.4904) nor in the control group (*P* ═ 0.4105). Previous studies have yielded conflicting findings regarding the impact of age on urinary ATP levels, with some studies indicating an increase in urinary ATP levels with age [14], while others suggesting a negative correlation [15] or finding no age-related effect at all [10, 11, 16]. The underlying reasons for these inconsistent outcomes remain unclear.

The method employed for urine sample collection in this study was non-invasive. This approach stands in contrast to some previous research, where fluid obtained during urodynamic examination was utilized [17, 18]. In our study, we posit that the selection of urine markers becomes inconsequential if urine is obtained through invasive means, as both the diagnosis and symptom severity can be accurately determined through cystoscopy and urodynamics. The ATP release from the urothelium is significantly affected by the bladder wall distension, with greater distension resulting in a higher ATP release. Recognizing this, to ensure accurate measurements, we implemented a correction method for urine volume. Patients were instructed to empty their bladders and subsequently provide midstream urine samples after consuming 250 mL of water over intervals of 10, 20, and 30 min. Considering that IC/BPS patients typically have smaller bladder capacities and feel urgency at lesser volumes than healthy individuals, we specifically instructed them not to urinate within 30 min after consuming water. This brief interval was chosen considering the instability of ATP in urine due to its degradation and short half-life. This could have potentially influenced the variations observed in urinary ATP concentrations between the samples collected in this study. To address the issue of ATP’s instability at room temperature, samples were immediately placed on ice. Adenylyl-imidodiphosphate was introduced to the samples within 30 min to prevent ATP degradation prior to testing. Moreover, this study excluded confounding factors, such as bacterial infections and malignancies, known to affect urinary ATP levels. It is important to acknowledge the variability potential in samples from volunteers depending on whether they were collected at the same location as the samples from the IC/BPS patients or at a different location. To mitigate potential discrepancies arising from sampling at different locations, we ensured that samples were collected in a uniform, tranquil treatment room for all participants. Additionally, the luciferin–luciferase reaction used in our study is highly susceptible to ion concentrations and potential drug concentrations in the urine. For instance, small variations in Mg^2+^ concentrations would alter the measured ATP concentration since magnesium is a crucial cofactor for this reaction. This reaction may also be susceptible to any drugs or drug metabolites that may be present in the urine, especially if not controlled in the inclusion/exclusion criteria (i.e., the patient is on blood pressure medication). While animal studies offer controls for drug effects by integrating them into the standard curve, used to convert luminometer readings to ATP concentrations, such controls are not possible in human patients. Therefore, alternative techniques, such as mass spectrometry, are necessary to further enhance the accuracy of future ATP measurements.

The underlying causes of IC/BPS remain elusive, leading to the absence of a universally accepted diagnostic approach. Typically, diagnoses rely on clinical assessments, which primarily encompass the exclusion of other prevalent conditions. Subsequently, a cystoscopy is conducted to detect the presence of Hunner’s ulcers or glomeruloid blebs [19]. However, many patients are hesitant to undergo cystoscopy due to its invasive nature, potential risks, pain, and financial implications. To address this issue, several biomarkers have been proposed for diagnosing IC/BPS, including growth factors, glycoproteins, chemokines, cytokines, and other relevant factors [20]. Nevertheless, there are several challenges that make it difficult for these biomarkers to be promoted on a large scale. One such challenge is the lack of specificity in the elevation of inflammatory cytokines and chemokines in the IC/BPS patients’ urine. Such inflammatory factors tend to increase whenever bladder inflammation occurs [20]. While the origin of urinary ATP is challenging to ascertain, existing studies indicate that it is predominantly derived from the urinary epithelium [21]. The urothelium of the bladder, when injured in IC/BPS, exhibits heightened vulnerability to both physical and chemical stimuli, leading to the release of mediators like ATP [22]. Additionally, the luciferin–luciferase bioluminescence assay for ATP is a straightforward, cost-effective, and exceedingly dependable technique for quantifying ATP levels.

The O’Leary–Sant ICSI/ICPI and VAS scores are widely recognized as reliable indicators of a patient’s disease severity [5]. Our findings revealed a significant correlation between urinary ATP concentration and IC/BPS symptom scores, which suggests that urinary ATP levels may serve as a valuable indicator of disease severity. However, our research did not involve comparing different treatment outcomes; therefore, there is no evidence to suggest that urinary ATP can predict responses to clinical interventions. Further investigation in longitudinal studies is warranted to explore this potential avenue.

Previous studies have primarily focused on investigating the diagnostic and therapeutic potential of urinary ATP in patients with OAB [23]. However, there is limited understanding of ATP’s role in IC/BPS. It is important to highlight that OAB and IC/BPS are distinct conditions, with the abnormal urinary epithelial barrier function being exclusive to IC/BPS patients, and not observed in those with OAB [24]. In contrast to IC/BPS, OAB does not present with suprapubic and lower abdominal pain symptoms. Additionally, the increased urinary frequency observed in OAB serves to prevent urinary incontinence, whereas in IC/BPS, it is a mechanism to alleviate pain [25]. Studies have demonstrated elevated urinary ATP levels among individuals diagnosed with OAB who also experience detrusor overactivity (DO) [23, 26]. Another study found elevated urinary ATP levels in individuals diagnosed with BPH who also exhibited DO [10]. Given these observations, it is proposed that DO may lead to heightened release of ATP within the bladder lumen.

IC/BPS patients exhibit elevated ATP levels in their urine, which increase with the severity of their symptoms. The strong positive correlation between urinary ATP levels and IC/BPS symptom scores, coupled with the high AUC of the ROC curve, indicates that urinary ATP could serve as a valuable non-invasive biomarker for IC/BPS patients. In contrast to the invasive and costly nature of cystoscopy, urinary ATP measuring is a simple, non-invasive, cost-effective, and reliable method that could potentially be used as an alternative approach for assessing IC/BPS. The integration of multiple distinct urinary factors has the potential to enhance the accuracy of IC/BPS diagnosis, differentiating it from non-inflammatory bladder conditions. The P2X3 receptor, as an ATP receptor, has been a target for drug development in the treatment of LUTS for decades [27]. While selective P2X3 antagonists, such as A-317491 and AF-353, have shown effectiveness in animal models of bladder dysfunction [28], they have not yet been used in clinical settings. This study paves the way for the potential clinical use of P2X3 antagonists in managing IC/BPS.

While this study focused primarily on the relationship between urinary ATP levels and IC/BPS, it did not account for other lower urinary tract conditions. In subsequent research, we intend to include diseases, such as UTI, BOO, and OAB, among others. By comparing the urinary ATP concentration across different diseases and over various time intervals, we aim to obtain more precise and compelling evidence. Additionally, the study’s sample size for IC/BPS patients was limited and exclusively comprised of female individuals. To validate these findings and delve deeper into potential biomarkers for IC/BPS, it is imperative to conduct extensive, multicenter studies. Such research endeavors will enhance the accuracy of disease diagnosis.

## Conclusion

In this study, urinary ATP levels were higher in women with IC/BPS compared to the control group. Furthermore, there was a strong positive correlation between these ATP levels and the disease symptom scores.
